# Challenges in recruiting children to a multidrug-resistant TB prevention trial

**DOI:** 10.5588/ijtld.21.0098

**Published:** 2021-10-01

**Authors:** S. Purchase, E. Batist, N. Mmile, S. Nkosi, J. Workman, N. Martinson, L. Fairlie, H. S. Schaaf, L. Choo, C. McGowan, A. M. Crook, J. A. Seddon, A. C. Hesseling

**Affiliations:** 1 Desmond Tutu TB Centre, Department of Paediatrics and Child Health, Faculty of Medicine and Health Sciences, Stellenbosch University, Tygerberg,; 2 Perinatal HIV Research Unit, University of Witwatersrand, Johannesburg,; 3 Wits Reproductive Health & HIV Institute, Faculty of Health Sciences, University of the Witwatersrand, Johannesburg,; 4 Department of Paediatrics and Child Health, Faculty of Medicine and Health Sciences, Stellenbosch University, Cape Town, South Africa;; 5 Institute of Clinical Trials and Methodology, MRC Clinical Trials Unit at University College London, London,; 6 Department of Infectious Diseases, Imperial College London, Norfolk Place, London, UK

**Keywords:** fluoroquinolone, TB-CHAMP, rando-mised controlled trial

## Abstract

**BACKGROUND:**

Recruitment to randomised clinical trials can be challenging and slow recruitment has serious consequences. This study aimed to summarise and reflect on the challenges in enrolling young children to a multidrug-resistant TB (MDR-TB) prevention trial in South Africa.

**METHODS:**

Recruitment to the Tuberculosis Child Multidrug-resistant Preventive Therapy Trial (TB-CHAMP) was tracked using an electronic recruiting platform, which was used to generate a recruiting flow diagram. Structured personnel questionnaires, meeting minutes and workshop notes were thematically analysed to elucidate barriers and solutions.

**RESULT:**

Of 3,682 (85.3%) adult rifampicin (RIF) resistant index cases with pre-screening outcomes, 1597 (43.4%) reported having no children under 5 years in the household and 562 (15.3%) were RIF-monoresistant. More than nine index cases were pre-screened for each child enrolled. Numerous barriers to recruitment were identified. Thorough recruitment planning, customised tracking data systems, a dedicated recruiting team with strong leadership, adequate resources to recruit across large geographic areas, and excellent relationships with routine TB services emerged as key factors to ensure successful recruitment.

**CONCLUSION:**

Recruitment of children into MDR-TB prevention trials can be difficult. Several MDR-TB prevention trials are underway, and lessons learnt from TB-CHAMP will be relevant to these and other TB prevention studies.

## RÉSUMÉ

**CONTEXTE:**

Il peut être difficile de recruter des patients pour des essais cliniques randomisés, et la lenteur du processus de recrutement peut avoir de graves conséquences. Cette étude avait pour objectif de résumer et d’analyser les défis liés à l’inclusion de jeunes enfants dans un essai de prévention de la TB multirésistante (MDR-TB) en Afrique du Sud.

**MÉTHODES:**

Le suivi du recrutement pour l’essai TB-CHAMP se fait à l’aide d’une plateforme de recrutement électronique, qui a été utilisée pour générer un diagramme de flux du recrutement. Les questionnaires structurés individuels, les comptes rendus de réunions et les notes des ateliers de travail ont été thématiquement analysés afin d’identifier les obstacles ainsi que des solutions.

**RÉSULTATS:**

Sur 3 682 (85,3%) cas index adultes de résistance à la rifampicine avec résultats avant dépistage, 1 597 (43,4%) ont rapporté n’avoir aucun enfant de moins de 5 ans au sein de leur foyer et 562 (15,3%) étaient des cas de mono-résistance à la rifampicine. Plus de neuf cas index ont été pré-dépistés pour chaque enfant inclus. De nombreux obstacles au recrutement ont été identifiés. Une organisation minutieuse du recrutement, des systèmes de suivi des données personnalisés, une équipe dédiée au recrutement bien encadrée, des ressources adéquates pour recruter sur de vastes zones géographiques et d’excellentes relations avec les services de lutte contre la TB de routine se sont révélés être des facteurs clés d’un processus de recrutement réussi.

**CONCLUSION:**

Il peut être difficile de recruter des enfants pour des essais cliniques de prévention de la MDR-TB. Plusieurs essais de prévention de la MDR-TB sont en cours, et les enseignements tirés de l’essai TB-CHAMP leur seront pertinents, ainsi qu’à d’autres études de prévention de la TB.

Modelling data suggest that 2 million children globally are infected with multidrug-resistant TB (MDR-TB) (i.e., *Mycobacterium tuberculosis* resistant to isoniazid [INH] and rifampicin [RIF] with or without resistance to other drugs), with 25,000– 32,000 children progressing to MDR-TB disease each year.^1,2^ The recent call to action by the UN High-Level Meeting included scaling up TB preventive therapy (TPT)^3^ and the WHO has emphasized the need for high-quality evidence from placebo-controlled trials to inform policy.^4^ The Tuberculosis Child Multidrug-resistant Preventive Therapy Trial (TB-CHAMP) is a community-based, multi-site cluster-randomised, placebo-controlled trial to assess the efficacy and safety of 24 weeks of daily levofloxacin in children aged <5 years exposed in their household to adults with MDR-TB. The trial is being implemented in South Africa and is the only trial exclusively investigating MDR-TB preventive therapy in young children.

Recruiting to randomised controlled trials (RCTs) can be challenging. A review of 440 trials between 2002 and 2008 found that only 55% met their planned sample size, and almost half (45%) had to request an extension.^5^ In a review of over 1000 RCTs, Kasenda and colleagues noted that a quarter were prematurely discontinued, with the most common reason for discontinuation being poor recruitment.^6^

There is a substantial body of literature highlighting common recruitment challenges to trials. Funding barriers include insufficient initial funding to cover recruiting costs and lack of additional funding for recruitment extention.^7^ Generally, the more rigorous the trial design, the more likely it is to encounter challenges. Strict inclusion and exclusion criteria, random allocation, blinding and use of placebo all make trials more challenging to enrol.^7–9^ Trials investigating infectious diseases in lower- and middle-income countries (LMICs) are usually conducted in poorer communities, with higher likelihood of migratory populations, violence, unemployment and substance abuse. Such trials are often conducted over large geographic areas, with poor public transport adding to recruitment complexity.

The lack of a sufficiently large pool of eligible participants to reach recruitment targets is another potential barrier. Muench’s Third Law states that the estimated number of potential participants that can be recruited should be divided by 10 to get a more accurate forecast.^10^ Schulz and colleagues use a rule of π by estimating how long recruitment will take and then multiplying by 3.14. For trials in LMICs, a multiplier of 2π is recommended.^10^

Barriers to recruitment for trial participants may include fear of adverse effects, mistrust regarding research, logistical issues, severe illness, language or cultural barriers and stigma around the disease being researched.^11^ In paediatric prevention trials, caregivers may be reluctant to expose their well children to an experimental regimen.^12,13^

Consequences of poor recruitment can be dire, and include trial abandonment, reduced statistical power, the need for supplemental funding which diverts resources from other trials, and frustration for funders, researchers, participants and communities. Slow accrual may delay identification of potentially lifesaving interventions.^10^ There is limited literature regarding challenges to recruitment in TB trials, and almost no data regarding TB prevention or paediatric trials. Few trials even report on recruitment details.^7^ Three MDR-TB prevention trials are currently underway (ACTRN12616000215426;^14^ NCT03568383;^15^ ISRCTN92634082^16^).

The purpose of this article is to describe challenges and solutions to recruitment in TB-CHAMP and provide practical lessons for investigators and other stakeholders to optimise recruitment to TB prevention trials, especially in children.

## METHODS

### Study design

**Figure 1:**
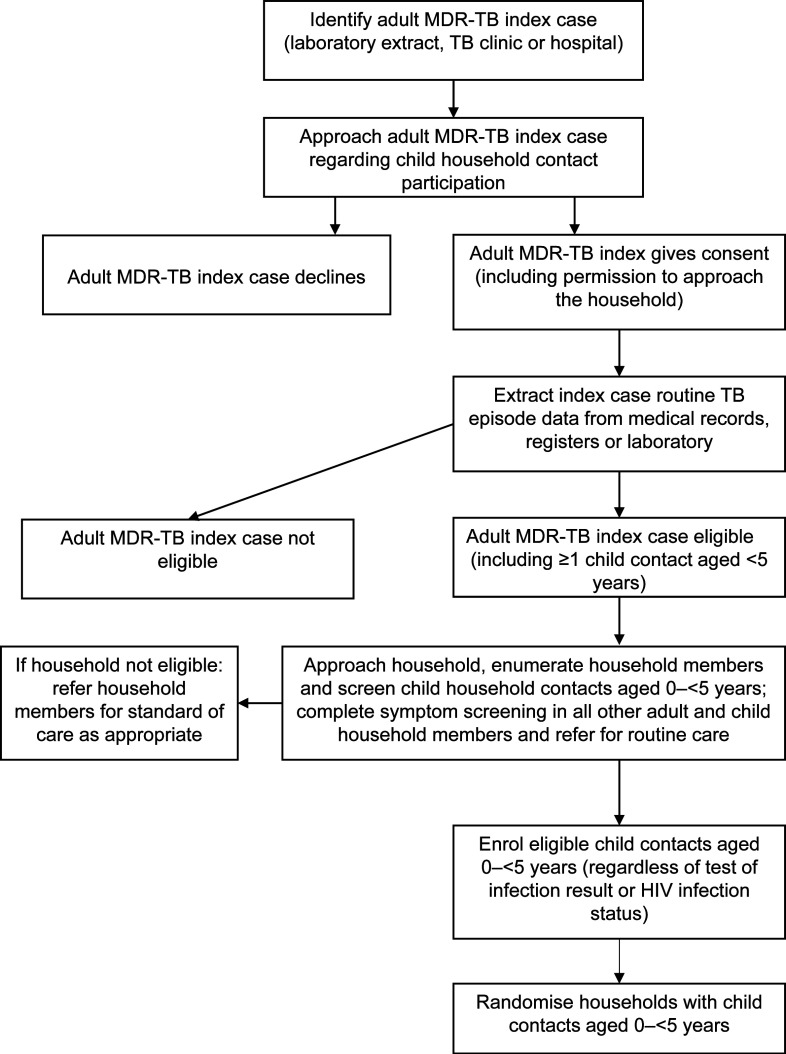
TB-CHAMP recruiting flow diagram. MDR-TB = multidrug-resistant TB; TB-CHAMP = Tuberculosis Child Multidrug-resistant Preventive Therapy Trial.

This was a mixed-methods sub-study, combining a quantitative analysis of recruitment flow using a CONSORT diagram with a descriptive qualitative, reflective process evaluation of trial recruitment. The data presented were collected between 1 September 2017 (trial opened) and 31 July 2019 (when recruitment was temporarily paused due to funding challenges). We summarise the recruitment flow, and our reflections on the recruitment process and the solutions put in place to address several challenges faced across sites. The COVID-19 pandemic has added multiple layers of complexity to this trial, which we will report on later. Figure 1 shows participant flow until child randomisation.

### Trial management and approvals

Stellenbosch University, Tygerberg, South Africa, is the trial sponsor, and trial management is supported by the Medical Research Council Clinical Trials Unit at University College, London, UK. The trial was approved by all relevant Research Ethics Committees or Institutional Review Boards in South Africa and the United Kingdom and by all required regulatory authorities. Index cases provided informed consent for their TB information to be captured and their household to be approached; caregivers provided informed consent for children’s participation.

### Setting

**Figure 2:**
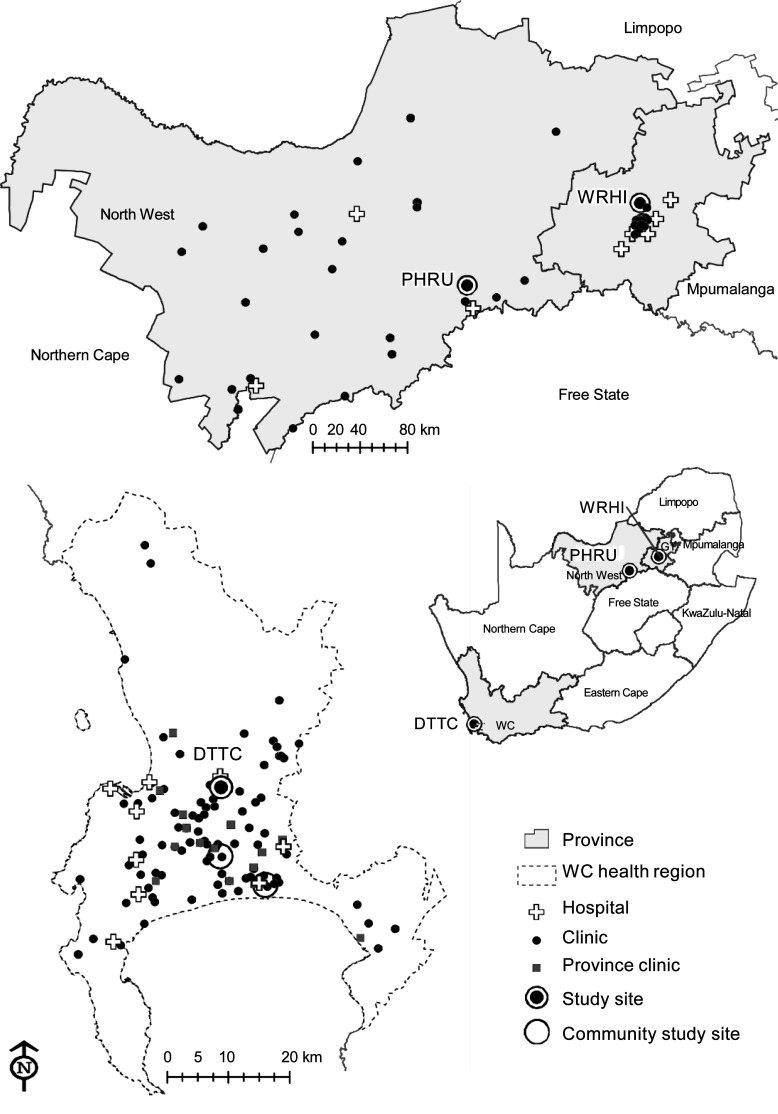
Location of South African sites conducting the TB-CHAMP study. PHRU = Perinatal HIV Research Unit, Soweto; WRHI = Wits Reproductive Health & HIV Institute, Johannesburg; GT = Gauteng; DTTC = Desmond Tutu TB Centre, Cape Town; WC = Western Cape; TB-CHAMP = Tuberculosis Child Multidrug-resistant Preventive Therapy Trial.

TB-CHAMP is being conducted at three South African research sites (Figure 2), all serving poor communities with a high burden of TB and HIV. Statistics from the Living Conditions Survey 2015 indicate that approximately half of South African adults live below the upper-bound poverty line, with 8% of children experiencing regular hunger.^17^

### Desmond Tutu TB Centre, Stellenbosch University, Tygerberg, South Africa

Recruitment took place from more than 100 primary healthcare (PHC) clinics and nine hospitals across metropolitan Cape Town, Western Cape Province. The study opened with permission to work in a subset of these facilities; further permissions were sought when recruitment was slower than anticipated. Community members are highly migratory, and families often separated, with children living in the more rural neighbouring Eastern Cape Province.

### Perinatal HIV Research Unit, Matlosana, South Africa

This peri-urban site is located at a general hospital, which serves as a referral centre for complicated MDR-TB patients from the North West Province. Recruitment took place from this hospital and three others with specialised MDR-TB units, and their referring PHC clinics. Most participants lived in relatively rural settings.

### Wits Reproductive Health and HIV Institute, Shandukani Research Centre, South Africa

Children were recruited from 20 PHC clinics and four hospitals in Johannesburg and surrounds. Participants were from a variety of urban communities.

In Cape Town when the study opened, it was policy in routine care to identify and screen MDR-TB contacts below 5 years, with an option to offer a three-drug preventive therapy regimen. There was limited MDR-TB contact tracing implemented at the Matlosana and Shandukani sites.

### Recruiting strategies

Adult MDR-TB index cases are identified from weekly data extracts of positive RIF-resistant Xpert^®^ MTB/RIF (Cepheid, Sunnyvale, CA, USA) results from the routine National Health Laboratory Service (NHLS), or from referrals by healthcare workers in routine TB services. Laboratory extracts were screened to assess initial eligibility. Permission was provided to the study teams to access this routine laboratory surveillance data to alert clinic personnel regarding potentially eligible index cases; the study team did not approach individual adult MDR-TB patients directly. Recruiting personnel assessed potential eligibility of the household and then arranged to obtain informed consent from the index case, typically at the clinic. Informed consent was also obtained from the caregiver/parent of the child participant. Potential child participants were screened at the trial sites for possible enrolment. This approach of starting with the adult MDR-TB index cases was necessary to ensure adequate identification and enrolment of child contacts, given the limited resources in routine TB services to conduct contact investigation in South Africa.

Each site developed its own recruiting plan and team structure, specific to the needs and challenges of its setting. Recruitment strategies were regularly revised with team structures altered and additional recruiters employed, with drivers functioning as recruiters and teams travelling up to 250 km from the research site to recruit participants.

Community advisory boards provided input into the study design, informed consent, and study messaging for the TB-CHAMP trial. The study team engaged regularly with routine healthcare services and study communities using posters, flyers and regular visits to all clinics, and by attending local training and dissemination meetings.

### Tracking the recruitment process

Pre-screening and screening processes were initially captured on paper-based logs. At two sites, online, shared spreadsheets were also used to track recruitment efforts. As numbers grew, these spreadsheets were found to be inefficient. A dedicated in-house recruiting platform ‘‘Mobilize’’ was developed to alleviate the administrative burden associated with recruitment tracking and to allow for accurate, up-to-date feedback for the recruiting team. Data were stored in a SQL database on secure servers with restricted access. Recruiters accessed the data and managed their recruitment strategies daily using a REDCap (Vanderbilt University, Nashville, TN, USA) user-interface, and study leaders were able to view summary statistics and trends in a Microsoft PowerBI dashboard (Microsoft, Redmond, WA, USA), embedded on the access-controlled Share-Point page.

### Data sources

Data were drawn from ‘‘Mobilize’’, paper-based logs and the TB-CHAMP clinical trial CACTUS database to understand patient flow and drop-offs. Information used to identify barriers and solutions came from several sources. Weekly site meetings and monthly team calls were held to discuss recruitment challenges. Two questionnaires were administered to study personnel during 2018. The first, developed with input from a socio-behavioural specialist, was a structured questionnaire completed by each site, including 29 questions regarding overall recruitment strategy and specific challenges faced while recruiting index cases and children. The second questionnaire was completed individually by each study team member, and asked team members to describe in free text and in detail the three biggest recruitment challenges experienced. Recruiting teams drew on daily study diaries, highlighting challenges encountered in the field. A full-day in-person workshop and brainstorming session including team members from all sites was held, and written input from all teams regarding recruiting challenges and solutions was solicited and collated. Content analysis of questionnaires and meeting minutes was completed, and major themes and sub-themes were identified, analysed and collated.

## RESULTS

### Recruitment outcomes

**Figure 3:**
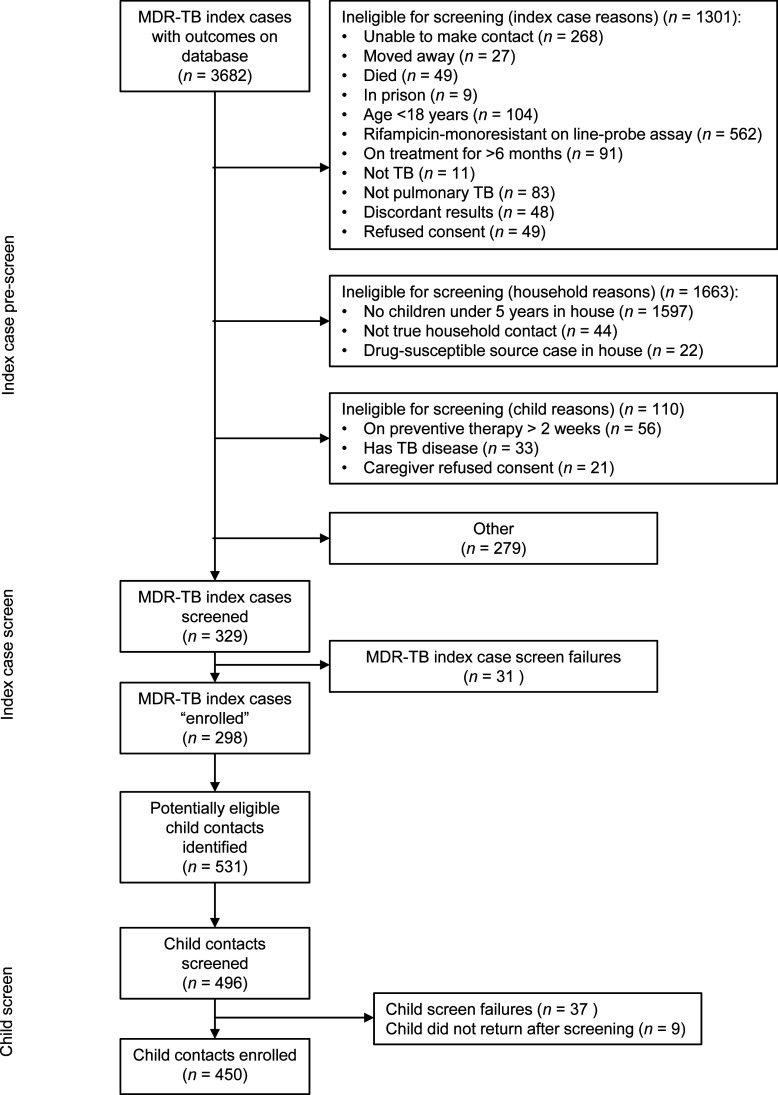
CONSORT diagram showing number of index cases pre-screened and screened, and number of children screened and enrolled on the TB CHAMP trial between 1 September 2017 and 31 July 2019. MDR-TB = multidrug-resistant TB; CONSORT = Consolidated Standards of Reporting Trials; TB-CHAMP = Tuberculosis Child Multidrug-resistant Preventive Therapy Trial.

There were 4,317 MDR-TB index cases identified overall over the 23-month recruiting period, mostly from the NHLS laboratory surveillance system (Figure 3). Of these, only 3,682 (85.3%) had prescreening outcomes allocated on ‘‘Mobilize’’ – prescreening outcomes were not captured initially at all sites. Of the 3,682 index cases with outcomes, 1,597 (43.4%) had no children under 5 years in the house and 562 (15.3%) were excluded due to RIF monoresistance on line-probe assay. This figure does not accurately reflect levels of RIF monoresistance, as some index cases with monoresistance may have been screened out for other reasons, and in some cases study teams could not wait for phenotypic INH drug susceptibility test results before excluding index cases. The team was unable to contact 268 (7.3%) index cases, despite multiple attempts. Forty-nine (1.3%) index cases had already died by the time the team made contact. Of 3,682 index cases with outcomes, 298 (8.1%) consented, allowing for the screening of 496 child contacts, with 450 children enrolled. Only 49 (1.3%) index cases and 21 (0.6%) caregivers refused consent; 1.5 children were enrolled from each index case recruited. Figure 3 shows the large number of index cases (9.6) needed to be pre-screened to recruit one child participant below 5 years of age.

### Recruiting challenges and solutions

Tables 1–3 give the challenges and solutions to recruitment identified by the trial teams at sites. These included participant-, study- and team/resource-related challenges.

**Table 1:** Participant challenges and solutions to recruiting for a large Phase 3 MDR-TB preventive therapy trial

Challenge	Possible solutions	Implemented?
Adult MDR-TB index case and/or caregiver		
Difficult to contact/locate (lack of contact details, migration, work schedule, illness, hospitalisation, incarceration)	Meet index case at clinic, drive together to home. Record multiple contact details. Work outside normal office hours. Obtain permission to recruit in hospitals	Yes
Illness (making consenting difficult), death	Be prepared to take consent in hospital, over multiple days. Allow relative of deceased index case to consent	Yes
Substance abuse (drugs, alcohol)	Be prepared to visit home on multiple occasions, especially early morning	Yes
Mistrust regarding research studies; low levels of research literacy	Well trained recruiters from local communities to take consent; active Community Advisory Board	Yes
Stigma, fear of rejection/eviction	Recruiters to discuss stigma at first contact. Use of unmarked cars and clothing. Option to use own transport to get to study site	Intermittently
Index case is a minor	Allow parent/legal guardian to provide consent	No
Child		
In foster care due to illness/hospitalisation of caregiver – unable to attend study visits	Take consent from parent/legal guardian. Arrange transport for child and foster parent for follow-up visits.	Yes
Caregiver		
No legal confirmation of guardianship	Assist family to obtain guardianship	Yes
Second parent refuses consent	Try to involve both parents in consent process	Yes

MDR-TB = multidrug-resistant TB.

**Table 2:** Study team/resource challenges and solutions to recruiting for a large Phase 3 multidrug-resistant TB preventive therapy trial

Challenge	Possible solutions	Implemented?
Short staffed, especially drivers	Budget carefully for support staff; train drivers as recruiters/vice versa	Over time
Lack of recruitment tracking system	Start study with good electronic recruitment tracking system; does not need to be complex	Over time
Dual roles (as recruiter and research assistant)	Carefully structure team and clarify roles – preferable to have a dedicated recruiting team	Over time
Lack of team leadership, clearly defined team structure	Recruitment team leader is key hire – motivated individual with good administrative, interpersonal skills	Over time
Communication between team members, multiple facilities, and study sites	User-friendly recruitment tracking system; WhatsApp groups; phones, data, airtime to all team members; dedicated study phone per study site; good internet connection at study sites	Over time
High staff turnover	Protocols/material in place for rapid training of new staff	Over time
Trial fatigue	Clear targets; staff incentives (meals, social events, small gifts)	Over time

**Table 3:** Study design and setting challenges and solutions to recruiting for a large Phase 3 MDR-TB preventive therapy trial

Challenge	Possible solutions	Implemented?
Randomised, placebo-controlled trial	Carefully explain rationale in simple language; meet regularly with routine healthcare team to discuss study rationale	Yes
Prevention trial	Carefully explain benefits of prevention	Yes
Long follow-up period	Explain rationale for follow-up period and stress that follow-up in routine care would be similar length	Yes
Time-consuming consent process	Use of recruiters to consent; drivers function as recruiters	Yes
Dual written consent (index case and caregiver) needed	Use of recruiters to consent; drivers function as recruiters	Yes
Index case criteria (adult, MDR-TB, diagnosed from sputum during last 6 months, rifampicin monoresistance excluded)	Data extract from laboratory very useful to identify newly diagnosed pulmonary TB adult index cases; careful follow-up and tracking to exclude rifampicin monoresistance	Yes
Child inclusion criteria (under 5, close household contact, preventive therapy <2 weeks)	Plan for large recruiting area; attempt to enrol children as soon as possible after index case is diagnosed	Over time
Potential duplication of work with routine care	Develop and pilot good communication tools between study and routine care	Over time
Long waiting times during study visits	Optimise clinic flow with available resources; participant appointments in different time slots; doctors start day by writing scripts to avoid pharmacy delays	Over time
Migrant population – moving regularly between homes, suburbs, provinces	Constantly update contact details; anticipate multiple attempts to make contact	Yes
Poor communities (homes difficult to locate, low level of education, comorbidities, substance abuse)	Make use of local knowledge, employ staff from local communities, simple language in study material	Yes
Violent communities	Staff safety is paramount: recruiters work in pairs, drivers accompany recruiters to homes, drivers with advanced driving skills, avoid potential hotspots	Yes
Over researched communities	Ensure excellent synergy and co-operation with other researchers in the area	Mostly
Large recruiting area, numerous clinics	Budget appropriately for transport costs	Over time
Health care worker concerns regarding study design	Face to face contact sessions with healthcare workers, as well as presentations at clinical meetings, forums; ready availability of supporting study documentation – simple, widely distributed	Yes
Over-worked health care workers in routine care; few referrals	Ensure referral to study is not onerous, study decreases workload for healthcare workers; promotional materials (mugs, pens, rulers) as reminders of study	Yes
Rapid turnover of health care workers in routine care	Regular updates, posters in each clinic, be prepared to explain study at each clinic visit	As far as possible
Conflicting trials	Large recruiting area, develop synergies, cross-referral	Yes
Hospitals and in-patients difficult to locate, often already discharged	Track index cases to local clinics using address details or laboratory system; knowledge of local geography and referral patterns crucial	Yes
Frequent unrest/strike action (cars mistaken for taxis)	Study vehicles to be clearly marked, using magnetic labelling (removable where stigma is a concern)	Yes

MDR-TB = multidrug-resistant TB.

### Participants

It soon became clear that study teams were dealing with households in crisis. Many index cases were hard to contact due to migration, hospitalisation or lack of a fixed address. Significant resources were expended for multiple clinic and home visits. Overall, 71% of index cases were either the primary caregiver or regularly cared for the child, and their illness impacted on family function. Anecdotally, perceived stigma related to TB and MDR-TB seemed prevalent, and study teams were often asked to visit homes in unmarked clothes and vehicles. Some individuals feared eviction from their homes should their MDR-TB status become public knowledge. Caregivers were hard to contact, often working outside of the home during the research teams’ working hours. However, once contacted, few index cases and caregivers refused consent. The use of well-trained research assistants from local communities, obtaining informed consent in simple language and in the participant’s preferred language was felt to be useful.

### Study design and setting

TB-CHAMP is a complex trial with a long follow-up period. The rationale of the trial needed to be carefully explained in appropriate language, and the informed consent process was time-consuming. Dual written consent was needed (from the index case and the caregiver), which prolonged the consenting process. Locating index cases was challenging, partly due to the decentralised policy for treatment of MDR-TB in South Africa. There were far fewer under-5-year-old child contacts than the 2 per household that we anticipated based on data from previous observational MDR-TB studies.^18,19^ RIF monoresistance impacted recruitment and rates were somewhat higher than the 8–12% levels anticipated from 2007–2009 surveillance data accessed when the study was designed in 2012.^20,21^ We did not consider the impact of discordant results at the time the study was designed, but these did impact recruitment - children living with adults with discordant INH susceptibility results were not enrolled due to the potential benefit of INH preventive therapy. At all sites, recruitment took place over large areas, in poor communities and with over-worked routine healthcare workers, who referred patients to multiple studies. Recruiters needed extensive local knowledge and spent a considerable amount of time gaining the trust of healthcare workers from numerous clinics and hospitals. In some instances, study vehicles were targeted for violent attacks as they were mistaken for taxis operating during strike action.

### Study teams

Recruitment was found to be very resource-intensive in terms of personnel and vehicles. This meant that teams needed to plan carefully. Personnel needed to have dual roles in some instances. Development of a recruitment tracking system was found to be very beneficial in enabling rapid communication between teams and in avoiding duplication of effort.

## DISCUSSION

Recruitment to prevention trials with rigorous study designs, which focus on infectious diseases and target highly vulnerable populations, is particularly chal-lenging, and researchers need to plan such trials carefully and in consultation with funders and community stakeholders.

Recruitment for the TB-CHAMP trial faced numerous obstacles. There were far fewer children under 5 in each household than we anticipated and almost half of all index cases were automatically excluded as a result. RIF monoresistance had more impact than expected, indicative of the evolving drug-resistant TB pandemic in South Africa. Although MDR-TB was sometimes confirmed later in index cases (based on culture of sputum samples using phenotypic INH DST), teams needed to act on molecular test results that showed INH susceptibility. Teams had to recruit over wide geographic areas and from many healthcare facilities, which was time- and resource-intensive. Locating MDR-TB index cases was difficult, and teams visited clinics multiple times to make contact. Initial recruitment tracking systems soon became cumbersome and did not facilitate rapid communication between team members at different locations. Direct referrals were fewer than anticipated, and laboratory data extracts became key to contacting index cases. Additional time and energy were invested in ongoing meetings with routine healthcare personnel regarding the trial. Contact tracing and recruiting in poor, sometimes violent communities where TB disease is stigmatised, meant extra resources were expended to ensure that study personnel could work safely and sensitively.

In a systematic review of discontinued trials, an overestimated prevalence of eligible participants was the most frequently reported reason for recruitment failure.^7^ Based on our experience, we recommend feasibility studies where possible, but at least the investment of time and resources on a detailed and careful recruitment plan, using current demographic and healthcare data to accurately estimate the prevalence of populations of interest. Nesting additional qualitative work to systematically investigate recruitment challenges and solutions is also recommended. We did not anticipate having to pre-screen 9–10 index cases for each child participant enrolled on TB-CHAMP. This had important implications for our study timeline and funding.

Recruitment strategies need to be dynamic and flexible. Key to this is a tracking/monitoring system that can provide rapid feedback.^22^ Recruiting teams need to be able to track referrals and attempts to contact participants and communicate these effectively. Teams must also be able to plan and strategize based on current data and projections. We found that a customised tracking database was useful and recommend that it be in place before a trial opens. Using trained research assistants and drivers as recruiters frees up clinical personnel. Strong leadership was required to ensure good recruitment and dynamic, organised individuals need to lead recruiting. Constant positive feedback was important, given the discouraging experiences while recruiting.

Our study had limitations. At one site, outcome data were incomplete, influencing our ability to provide a true reflection of reasons why index cases screened out. Challenges and solutions listed are the combined opinions of all study personnel members but were not systematically collected. Nevertheless, we have learned invaluable, practical lessons which are relevant to other TB prevention trials.
